# Supplementary material for: Prevention of child wasting: Results of a Child Health & Nutrition Research Initiative (CHNRI) prioritisation exercise
Source: PLoS One. 2020 Feb 12;15(2):e0228151. doi: 10.1371/journal.pone.0228151 (PMC7015423; doi:10.1371/journal.pone.0228151)
Supplement: S1 File — (DOCX) [file pone.0228151.s001.docx]

**Wasting Prevention Research Prioritisation – Scope, Definitions and Context**

Wasting is widely referred to as an acute condition compared to another manifestation of undernutrition. Stunting is often referred to as chronic malnutrition. Wasting can be relatively rapid in onset and resolution on an individual level, hence, the term acutely malnourished (divided into moderate and severe) is used to describe children who are wasted. However, the term ‘acutely malnourished’ also includes children suffering from other relatively rapid onset manifestations of undernutrition; kwashiorkor (characterised by nutritional oedema), small mid-upper arm circumference (MUAC), (characterised by a loss of muscle and fat tissue) and low weight-for-age (WFA) due to the evidence of their relationship with mortality in infants and children and because WFA and MUAC partly reflect degrees of wasting on an individual level. These measures are therefore all considered as outcomes of interest for this RP although they are outside of the standard WHO definition of ‘wasted’ (*WHO* 2009).

It is important to note that wasting (as with other manifestations of malnutrition) is a process which may lead to a child being wasted and resulting in functional problems (altered body composition and diminished physical and mental function’ - Cederholm *et al.*, 2015) Anthropometric measures and indices are only proxies for this process and were mainly designed to be applied to populations rather than individuals. Therefore, this RP considers two dimensions of prevention; *primary prevention* (prevention of any degree of wasting including from the initiation of the process that leads to an infant/child becoming wasted) and *secondary prevention* (prevention of worsening degrees of wasting i.e. from moderate to severe which lead to progressively high mortality risk). It also considers *wasting in individuals* as well as high and/or *persistent prevalence* and incidence of wasting on a population level. It does not consider prevention of relapse to wasting as this is defined as a deterioration immediately post treatment (within 3 months) and therefore an issue of ensuring complete and effective treatment of the condition rather than of prevention approaches.

Finally, in recognition of recent advances in understanding of the relationship between wasting and stunting (ponderal and linear growth) the EGrecommend that any research with intended outcomes on wasting or other relatively acute forms of undernutrition also explore patterns in, or effects on linear growth/degree of stunting.

See below table outlining the ‘context’ for this CHNRI RP exercise. Note that although we are following the formal CHNRI approach, the EG recognise that the research questions of interest do not lie solely in the health domain, but will span non-health related problems such as peoples livelihoods, women’s empowerment, the sanitary environment etc.

| Area | Notes from CHNRI methods | Context |
| --- | --- | --- |
| Population of interest | Who’s nutrition problems are being addressed through the priority setting | Direct  Infants <6m  Children 6-59m  Indirect (1000 days and beyond)  Adolescent girls  Women  Caretakers  Populations/ communities affected by persistent wasting i.e. > 15% prevalence over many years |
| The disease burden of interest | What is known about the burden of disease, disability, and death that will  be addressed by supported nutrition research | Global prevalence and yearly incidence of wasted children (using prevalence = 50.5 million children 0-59m of age wasted)  Global prevalence & yearly incidence of severe wasting (using prevalence = 17 to 19 million children 0-59m of age wasted)  Global prevalence & yearly incidence of small MUAC, Kwashiorkor (no global estimate but regions of southern and eastern Africa suggest kwashiorkor accounts for 50–70% of cases of severe malnutrition), low weight-for-age  Mortality burden associated with wasting (based on prevalence) = 875,000 deaths (Lancet estimates)  Yearly Disability-Adjusted Life-Years (DALYs) attributable to wasting/acute malnutrition globally |
| Geographic limits | Boundaries in terms of space, which may be global, regional, national, sub–national, etc. | LMIC countries with a burden of wasted infants/children 0-59. /including those with persistent high levels of wasting (GAM>15% over a number of years (e.g. 5 years) despite significant humanitarian intervention).  In some cases research to be carried out in high income countries may be included where there is direct relevance to understanding how to prevent wasting in LMIC.  Research that focusses on sub-national, national, regional or global level. |
| Timescale | Level of urgency, i.e., in how many years are the first results of the proposed research expected  (they may be defined as reaching the endpoints of the research process, or translating and implementing them, or achieving detectable disease burden reduction). | To achieve measurable results in infants/children (or concrete potential for future results) in the above disease burdens of interest within 5 years |
| Preferred style of investing with respect to risk | Investment strategy in research with respect to risk preferences; it defines whether most of the funding would support a single (or a few) expensive high–risk research ideas (e.g., vaccine development), or will the risk be balanced and diversified between many research options which will have different levels of risk and feasibility. | Research which will be diversified across different contexts where wasting is a problem (Asia, Africa, Caribbean etc.) and will have different levels of risk and feasibility. It will encompass a range of research options/areas covering infants, children and potentially adolescent girls and women. Some will be in Fragile and Conflict Affected States (FCAS) contexts where access may be an issue. |

**Important information on the scope of this exercise**

*Time frame:* Questions have been included in this research prioritisation exercise (RP) that could produce results within a *five-year research period*.

*Target group:* The questions in this RP refer primarily to *infants and children aged 0 to 59 months living in low and middle-income countries (LMIC).* In some cases, research to be carried out in high income countries has been included where there is direct relevance to understanding how to prevent wasting in LMIC. Some questions also concern those groups indirectly affected by wasting in this age group (including adolescent girls, caretakers and communities where wasting is prevalent).

*Focus on prevention:* The questions are focussed on wasting prevention *and not on treatment* (which has been comprehensively covered in previous global research prioritisation exercises).

*Anthropometric outcomes of interest:* Wasting (characterised by low weight-for-height/length) can be relatively rapid in onset and resolution on an individual level, hence, the term acutely malnourished (divided into moderate and severe) is often used to describe children who are wasted. Other forms of acute undernutrition (characterised by the presence of bilateral oedema, low mid-upper arm circumference or low weight-for-age) are also considered due to the evidence of their relationship with mortality in infants and children. These measures are therefore all outcomes of interest for this RP despite being outside of the standard WHO definition of ‘wasted’ (WHO and UNICEF 2009).

*Definition of prevention:* Wasting is a process which may or may not lead to a child becoming wasted. Anthropometric measures and indices are only proxies for this process, designed mainly to be applied to populations rather than individuals. Therefore, this RP considers two dimensions of prevention: primary prevention (prevention of any degree of wasting including from the initiation of the process that leads to an infant/child becoming wasted) and secondary prevention (prevention of worsening degrees of wasting).

*Relationship between wasting and stunting:* Due to recent advances in understanding of the relationship between wasting and stunting, the RP questions also emphasise the importance of not researching wasting in isolation from other outcomes of undernutrition, stunting in particular.
