# Supplementary material for: Prevention of child wasting: Results of a Child Health & Nutrition Research Initiative (CHNRI) prioritisation exercise
Source: PLoS One. 2020 Feb 12;15(2):e0228151. doi: 10.1371/journal.pone.0228151 (PMC7015423; doi:10.1371/journal.pone.0228151)
Supplement: S1 Table — (DOCX) [file pone.0228151.s002.docx]

**Table 1: Research questions ranking according to the overall research priority score (RPS)**

| Research question | No. | Rank | Group | A | E | D | F | RPS | AEA | N |
| --- | --- | --- | --- | --- | --- | --- | --- | --- | --- | --- |
| What is the impact of management of at-risk mothers & infants <6 months interventions in preventing wasting/acute malnutrition in the >6m old? | 17 | 1 | Description | 97.5 | 96.7 | 93.9 | 95.9 | 96.0 | 93.1 | 122 |
| What is the impact of growth failure during the first 6 months of life on experience of wasting/acute malnutrition after 6 months of age? | 14 | 2 | Description | 96.3 | 88.0 | 88.9 | 89.3 | 90.6 | 86.4 | 117 |
| How can existing interventions (e.g. growth monitoring, integrated management of childhood illness (IMCI)) better detect and support children (0-59 months) who are failing to thrive/faltering (i.e. those at-risk, not just those already below a z-score threshold)? | 29 | 3 | Development | 91.4 | 90.1 | 89.8 | 87.8 | 89.7 | 85.6 | 131 |
| What are effective and cost-effective approaches to target the highest risk infants and children 0-59 months (e.g. children with concurrent wasting/acute malnutrition and stunting, children <24 months, etc) for interventions (food or non-food) to prevent wasting/acute malnutrition? | 25 | 4 | Delivery | 93.3 | 89.4 | 85.4 | 88.4 | 89.1 | 84.7 | 129 |
| What measures (anthropometric or non-anthropometric) or combinations of measures best identify individual infants and children (0-59 months) by age/sex at most risk of death/other adverse outcomes associated with wasting/acute malnutrition? | 2 | 5 | Description | 91.9 | 89.2 | 87.4 | 85.5 | 88.5 | 82.8 | 128 |
| What is the role of pre-pregnancy maternal factors (age, health status, nutritional deficits, psychological factors etc.) in determining risk of being born with a low birth weight, low weight-for-length, low mid-upper-arm circumference, premature or small for gestational age? | 15 | 6 | Description | 96.7 | 87.6 | 86.4 | 83.2 | 88.5 | 83.2 | 122 |
| What measures (anthropometric or non-anthropometric), or combinations of measures, best identify individual infants and children (0-59 months) by age/sex at most risk of wasting/acute malnutrition? | 1 | 7 | Description | 92.3 | 88.2 | 89.0 | 81.3 | 87.7 | 82.0 | 128 |
| What are effective and cost-effective approaches to integrating wasting/acute malnutrition prevention efforts into health systems (i.e. human resource capacity, financing, supplies and supply chain, etc)? | 21 | 8 | Delivery | 86.0 | 89.9 | 84.3 | 89.5 | 87.4 | 81.0 | 128 |
| What impact can effective wasting/acute malnutrition prevention interventions/approaches have on levels of stunting (and concurrent wasting and stunting) and vice versa? | 18 | 9 | Description | 89.5 | 85.5 | 85.0 | 87.2 | 86.8 | 79.5 | 125 |
| How does being born prematurely and/or with foetal growth restriction impact on wasting/acute malnutrition at birth and throughout the first 5 years of life, by sex? | 13 | 10 | Description | 93.0 | 81.6 | 83.8 | 86.5 | 86.2 | 80.2 | 122 |
| What combinations/interactions of risk factors (and their prevalence), by age/sex, lead to wasting/acute malnutrition in a given context? | 10 | 11 | Description | 90.9 | 84.6 | 86.5 | 82.7 | 86.1 | 78.5 | 127 |
| What approaches/operational models of community engagement are effective in preventing wasting/acute malnutrition in a given context? | 24 | 12 | Delivery | 87.1 | 90.8 | 82.4 | 84.1 | 86.1 | 79.2 | 126 |
| What are the relative benefits, risks and cost effectiveness of food-based, product-based, or combined food and product-based approaches in wasting/acute malnutrition prevention? | 27 | 13 | Development | 89.2 | 86.2 | 83.7 | 84.4 | 85.9 | 79.8 | 122 |
| What is the contribution of episodes of wasting/acute malnutrition to stunting (in a given context), and vice versa? | 12 | 14 | Description | 91.3 | 82.6 | 81.8 | 87.4 | 85.8 | 78.4 | 123 |
| What are the effective approaches/methods (e.g. addition of indicators into national information systems) to coordinate and standardise the collection of routine monitoring data and its use for planning and evaluating wasting/acute malnutrition prevention interventions? | 22 | 15 | Delivery | 91.1 | 84.0 | 83.6 | 83.7 | 85.6 | 80.1 | 123 |
| What should an intervention package be composed of and what coverage should it achieve in order to reduce prevalence/incidence of child <5yrs wasting/acute malnutrition in a given context? | 23 | 16 | Delivery | 84.0 | 89.3 | 80.6 | 88.0 | 85.5 | 78.2 | 125 |
| What are the impacts of wasting/acute malnutrition with/without stunting on mortality by sex/age group? | 7 | 17 | Description | 95.2 | 82.7 | 85.1 | 77.6 | 85.1 | 80.7 | 125 |
| What measurements and thresholds can best estimate population prevalence and incidence of wasting/acute malnutrition in children from birth to 5 years, by age and sex (e.g. weight-for-age, weight-for-height, mid-upper arm circumference, concurrence of wasting and stunting (WaSt) in their severe and moderate forms) in a given context? | 4 | 18 | Description | 90.2 | 83.7 | 84.0 | 82.5 | 85.1 | 79.8 | 126 |
| What proportion of untreated moderately wasted/acutely malnourished children deteriorate further to severe wasting/acute malnutrition or spontaneously recover, by sex/age? | 6 | 19 | Description | 82.0 | 82.1 | 81.7 | 90.8 | 84.2 | 77.6 | 125 |
| What programmatic or project-based innovations (across all sectors / multi-sectoral) have led to prevention of wasting/acute malnutrition in a given context? | 38 | 20 | Discovery | 83.3 | 85.7 | 81.6 | 84.6 | 83.8 | 75.6 | 127 |
| How is it best to estimate annual incidence and year-on-year trends in wasting/acute malnutrition in a given context (e.g. stable populations, acute shocks) by sex/age group including accounting for spatial and temporal patterns, and population growth? | 5 | 21 | Description | 83.3 | 83.3 | 82.4 | 85.8 | 83.7 | 74.4 | 120 |
| What methodologies can best identify determinants of and pathways to wasting/acute malnutrition in a given context (including seasonal patterns) in order to understand their inter-relationships and identify priority risk factors or combinations of risk factors? | 9 | 22 | Description | 87.6 | 84.2 | 77.9 | 85.0 | 83.7 | 75.5 | 120 |
| Where do the determinants of stunting and wasting/acute malnutrition overlap? | 11 | 23 | Description | 87.2 | 79.8 | 81.8 | 85.7 | 83.6 | 75.2 | 122 |
| What is the additional benefit (effectiveness, cost-effectiveness) of nutrition-sensitive interventions (e.g. water, sanitation and hygiene (WASH)) over nutrition-specific interventions alone and vice-versa in wasting/acute malnutrition prevention? | 28 | 24 | Development | 87.4 | 80.3 | 79.8 | 81.4 | 82.2 | 75.0 | 129 |
| How can policies which better support optimal infant and young child feeding have an effect on prevention of wasting/acute malnutrition? | 20 | 25 | Delivery | 88.3 | 81.6 | 79.5 | 78.7 | 82.0 | 75.8 | 129 |
| What measures (anthropometric or non-anthropometric), or combinations of measures, best identify the earliest point at which a child has started a downwards trajectory towards becoming wasted/acutely malnourished? | 3 | 26 | Description | 83.8 | 81.3 | 76.7 | 85.7 | 81.8 | 72.5 | 119 |
| How do current policies and governance/coordination systems promote an enabling environment for multi-sectoral approaches (and nutrition-sensitive programming) to prevent wasting/acute malnutrition, and can they be improved? | 19 | 27 | Delivery | 82.8 | 81.6 | 76.5 | 85.2 | 81.5 | 73.7 | 122 |
| How do the multiple uses and management of water resources at regional, community and household levels impact wasting/acute malnutrition? | 39 | 28 | Discovery | 84.6 | 81.0 | 79.8 | 80.3 | 81.4 | 75.4 | 119 |
| What policy processes or changes (e.g. in health system) can be linked to promoting an enabling environment for nutrition-sensitive/nutrition-specific approaches that have led to prevention of wasting/ acute malnutrition? | 40 | 29 | Discovery | 77.2 | 81.3 | 78.5 | 87.2 | 81.0 | 72.6 | 125 |
| What are the longer-term impacts of wasting/acute malnutrition on immune function, gut health (including microbiome), body composition and risk of obesity/non-communicable diseases (NCDs) by sex/age group? | 31 | 30 | Discovery | 84.5 | 74.6 | 73.0 | 87.2 | 79.8 | 70.9 | 121 |
| What are the longer-term impacts of wasting/acute malnutrition on disability (according to World Health Organization definition https://www.who.int/topics/disabilities/en/) by sex/age group? | 8 | 31 | Description | 85.7 | 74.4 | 73.7 | 85.4 | 79.8 | 69.1 | 120 |
| Which physiological factors explain the multiplicative effect of wasting/acute malnutrition and stunting on mortality? | 36 | 32 | Discovery | 84.7 | 77.8 | 72.9 | 82.9 | 79.6 | 68.0 | 117 |
| What is the impact of body shape and body composition on weight-for-height as a measure of nutritional status in infants and children (by sex and age)? | 33 | 33 | Discovery | 86.9 | 75.4 | 75.0 | 76.8 | 78.5 | 72.0 | 114 |
| What is the effect of probiotics and prebiotics on the microbiome (in particular through breastfeeding) and the potential role in preventing wasting/acute malnutrition? | 32 | 34 | Discovery | 84.7 | 77.5 | 70.8 | 80.6 | 78.4 | 68.6 | 108 |
| What are the essential nutrition-specific and nutrition-sensitive interventions, linkages and timings required within a package for it to be effective and cost-effective in preventing a decline in nutritional status (including wasting/acute malnutrition) in a given population or context (such as populations with high persistent wasting/acute malnutrition, environmental variability, fragile and conflict affected states)? | 26 | 35 | Development | 73.8 | 81.8 | 71.4 | 85.9 | 78.2 | 70.0 | 128 |
| To what extent can epigenetic mechanisms explain the association between maternal environmental factors in the first 1000 days (nutrition, stress, pollutant exposure, etc.) and later risk of adverse growth outcomes in childhood (wasting / acute malnutrition and stunting)? | 34 | 36 | Discovery | 82.3 | 75.9 | 66.2 | 86.2 | 77.7 | 68.0 | 109 |
| Can foods containing probiotics and prebiotics be effectively used in the prevention and treatment wasting/acute malnutrition and/or stunting? | 37 | 37 | Discovery | 85.0 | 75.0 | 69.9 | 80.0 | 77.5 | 67.7 | 110 |
| Is breastmilk quality/quantity a risk factor for wasting/acute malnutrition by child sex/age? | 16 | 38 | Description | 83.3 | 73.3 | 76.5 | 74.6 | 76.9 | 68.9 | 118 |
| What are the mechanisms that explain the different risk of wasting/acute malnutrition in girls and boys? | 35 | 39 | Discovery | 83.5 | 71.8 | 70.2 | 81.8 | 76.8 | 65.3 | 121 |
| What is the effect of the microbiome and environmental enteric dysfunction on energy and nutrient absorption from food (including supplementary food) and what are the implications for defining nutrient requirements to prevent wasting/acute malnutrition? | 30 | 40 | Discovery | 75.2 | 74.8 | 70.5 | 84.5 | 76.3 | 65.6 | 110 |

No= question number ; A = answerability; E = efficacy; D = deliverability; F = fills a gap; RPS = overall research priority score; AEA = average expert agreement; N = number of respondents
