# Supplementary material for: Prevention of child wasting: Results of a Child Health & Nutrition Research Initiative (CHNRI) prioritisation exercise
Source: PLoS One. 2020 Feb 12;15(2):e0228151. doi: 10.1371/journal.pone.0228151 (PMC7015423; doi:10.1371/journal.pone.0228151)
Supplement: Attachment — Submitted filename: CHNRI for Wasting Paper = Comments.docx [file pone.0228151.s004.docx]

CHNRI for Wasting Paper

This paper fills an important knowledge gap on research priorities to prevent wasting in children.

I have some suggestions and comments on the paper, described below:

1. I like how the DDDD was put into the context of wasting for the reader – this makes it more accessible.
2. The authors describe randomising each research option with the DDDD categories to limit the bias that these categories and the order of appearance of questions have on the scores. Were the DDDD categories themselves randomised? If not, which categories were presented first? What impact might this have had on scoring?
3. The AEA formula provided is incorrect. There are 4 questions, not 12 (the formula used is for a 4 criteria exercise, with 3 sub-questions per criteria). The correct formula is below. Additionally, below it should say: 4 is the number of possible scores for each research option across criteria.’

$$AEA= \frac{1}{4}X \sum_{q=1}^{4} \frac{N(number of scorers with most frequent}{N(number of scorers)}$$

4. On ethics, while CHNRI did not traditionally require ethics, ethics board are getting stricter and this is not always the case. One better way to justify not going through ethics is to have respondents be co-authors (with group authorship), thereby not having them be participants in the traditional sense, and arguing that it is akin to an organisational strengthening exercise
5. It would be helpful to state in your summary of participants how quickly they were aborted (e.g. since only 35% of the surveys were completed, were 60% aborted after completing one question? Or were they mostly complete?). You could also define 80% or more as a complete (enough) survey, and say 35% had 100% completeness, but X% had completeness for the purposes of analysis. And exclude any which were very incomplete (from analysis and summary of participants). This may require a more substantial edit, and could change the results depending on the cut-off you use for a ‘complete’ survey.
6. It’s worth noting that the range of AEA is very high for CHNRI. You discuss this in relation to people belonging to similar groups, etc., but is there any relationship between the rank and AEA and the number of scorers per each RQ and the AEA?
7. As a preference, I’d move the rank column to the left of the RQ column (so the RQs are numbered on the left in the table)
8. There appears to be a large number of scorers for the top 10 RQs. Is this because more people scored the highest ranked RQs? Could this be due to the DDDD randomisation (e.g. certain categories being presented first), given only 35% of respondents answered the entire survey? May be useful to discuss this.
9. I think graphing the AEA is less useful than discussing surprising results – e.g., where there any surprising results within criteria for the top 10? Bottom 10? Any places where RQs seemed to score low across the board? Some that scored high that was a surprise? Anything that’s supported by context?
10. There were minor typos throughout the paper. It would be beneficial to have a close read to find and fix these, prior to publication, as I know PLoS does not provide copy-editing. Examples of typos found include:
    a. Differences in font/font size in some places in the paper
    b. Incorrect use of ‘;’
    c. Some spaces missing, particularly before a citation
    d. On pg. 11, survey participants (e.g. not surveys) and later, participants should be plural
    e. On pg. 18, ‘selection biases can occur’ should be followed by a period not colon
    f. Some typos in acknowledgements section
